# Editorial: Immunosenescence after sepsis

**DOI:** 10.3389/fimmu.2023.1177148

**Published:** 2023-03-10

**Authors:** Xuan Lu, Yuan-Qiang Lu

**Affiliations:** ^1^ Department of Emergency Medicine, The First Affiliated Hospital, School of Medicine, Zhejiang University, Hangzhou, Zhejiang, China; ^2^ The Key Laboratory for Diagnosis and Treatment of Aging and Physic-chemical Injury Diseases of Zhejiang Province, Hangzhou, Zhejiang, China

**Keywords:** sepsis, immunosuppression, immunosenescence, Treg cells, cytokines, myeloid-derived suppressor cell

Patients with sepsis could be in a state of immune disorder in which excessive inflammation and immunosuppression may occur simultaneously (1). Patients experience a dysregulated host response to infection that can lead to a life-threatening organ dysfunction. Profound and sustained immunosuppression was thought to be responsible for susceptibility to secondary infection and increased mortality (2). Immunosenescence was initially thought to be a series of age- related changes in the immune system. Subsequently, it was found that low-grade chronic inflammation in age-related diseases, such as cancer, coronavirus disease 2019 (COVID-19), neurodegenerative diseases, and sepsis, may contribute to immunosenescence (Wang et al.). This Research Topic explored the potential mechanism of immunosuppression in sepsis, including immunosenescence, searched for immune-related prognostic markers, and discussed the clinical application of immunosuppressive drugs. Our Research Topic gathered 5 articles (including 3 original research articles, 1 review, and 1 systematic review) that could improve the understanding of immunosuppression in sepsis.

## Immune cell senescence related to organ dysfunction in sepsis

Immune cell senescence is manifested by a decrease in number and function of immune cells (3). Lu et al. summarized the possible mechanisms of immunosenescence and parenchymal organ damage. Sepsis-induced oxidative stress may lead to a decrease in cellular telomerase activity and telomere shortening. Age-related methylation in the nuclei and mitochondrial DNA is a kind of reprogramming of immune cells, which is a significant manifestation of immunosuppression. Otherwise, the inhibition of immune checkpoints, such as programmed death 1 (PD-1) and cytotoxic T-lymphocyte–associated antigen 4 (CTLA-4), is considered as a method to improve immunosuppression (4). However, the relevance of immune checkpoint regulation to immunosenescence is still worth testing.

The effects of immunosenescence on solid organs after sepsis have been observed in the lungs, brain, heart, kidneys, and liver. Senescent immune cells in the lungs could lead to decreased cell proliferation and impaired cytokine secretion. There’s a high susceptibility to lung disease. Immunosenescence could disrupt the intrinsic barrier of the brain and release pro-inflammatory mediators. On the other hand, immunosenescence after sepsis may lead to local myocardial ischemia or infarction secondary to coronary artery disease and renal dysfunction. Although liver damage appears in the early stages of sepsis, the effect of immunosenescence on liver injury deserves further investigation.

## Inhibition of myeloid-derived suppressor cell amplification in sepsis may improve patient outcomes

Lu et al. also summarized the role of MDSCs in sepsis. MDSCs can be amplified and activated by 1) emergency bone marrow generation driven by exogenous stimuli such as infection; 2) Pathological myeloid activation induced by the release of damage-associated molecular patterns (DAMPs) or pathogen-associated molecular patterns (PAMPs) in organ injury and secondary infection. In sepsis, MDSCs inhibited the function of dendritic cells (DCs) and macrophages (Sehgal et al.). At the same time, MDSCs reduced the diversity of NK cells. MDSCs inhibited Th1 response but induced Th2 and regulatory T (Treg) cells (5). Therefore, MDSC amplification is considered as a marker of immunosuppression in sepsis.

MDSCs can be divided into two major subpopulations: polymorphonuclear MDSCs (PMN-MDSCs) and monocyte MDSCs (M-MDSCs). Feng et al. explored the regulatory effect of continuous renal replacement therapy (CRRT) on MDSCs subpopulations in pediatric sepsis. Their study represented a significant increase in PMN-MDSCs in children with sepsis compared to healthy children. The proportion of PMN-MDSCs in survivors on CRRT 7^th^ day showed a decreasing trend in survivors. The percentage of CD8^+^ cells recovered and increased on CRRT 7^th^ day. The possible relationship between CRRT reduced MDSCs levels and T cell activation could be investigated. They also found that the reduction of PMN-MDSC amplification may be related to the reduction of IL-6 levels in patients treated with CRRT.

## Sepsis prognosis can be predicted by immune genes

The search for immune markers is helpful for the treatment and prognosis prediction of sepsis. The research of Liu et al. pointed a novel immune gene that could be valuable for predicting the outcome of sepsis patients. Through analysis of public databases and validation of the sepsis patient cohort, FCGR2C was considered to have good predictive efficacy. FCGR2C was also found to be closely related to a variety of immune cell functions. Cytotoxic lymphocytes were significantly increased in the sepsis death group, and FCGR2C was negatively correlated with cytotoxic lymphocytes. As a biomarker of bacterial infection, the role of red blood cell distribution width (RDW) in sepsis was systematically reviewed by Wu et al. Finally, RDW is considered to be a feasible and sensitive biomarker for predicting mortality in patients with sepsis.

## The prognostic model of sepsis can guide the clinical use of medications

The differentially expressed genes (DEGs) associated with sepsis can be found by single-cell RNA sequencing (scRNA-seq) and transcriptome RNA-seq. He et al. built a prognostic model of sepsis with DEGs including CCL5, HBD, IFR2BP2, LTB, and WFDC1. The risk prediction model and enrichment analysis showed that there was a difference in the abundance of immune cells in low - and high-risk sepsis patients. Treg cells, CD4 memory activated T cells, resting NK cells, M0 and M2 macrophages were higher in the high-risk group than in the low-risk group. They further analyzed immunotherapeutic targets of commonly used immune-related drugs in sepsis, such as immunostimulatory drugs, immunostimulatory cytokines, and immunosuppressants. Nine target sites were identified, of which IL-7 is considered to be the most promising immunotherapy.

## Summary

The immune status of sepsis is very complex and results from the combination of excessive inflammatory response and immunosuppression. Our Research Topic was ranging from mechanisms of immunosuppression, immune gene-related prognostic indicators, to monitoring clinical drug targets in sepsis. Immunosenescence is a novel study of sepsis immunity. We can learn from its mechanism of action in tumor microenvironment to further explore its influence on sepsis. As representative cells of sepsis immunosuppression, MDSCs had been reported for the first time to show proliferative inhibition in pediatric sepsis by CRRT treatment. The discovery of immune-related genes may be complementary to predict the prognosis of sepsis. Our Research Topic provides a new idea to study the mechanism of sepsis immunosuppression and paves the way for the clinical transformation of sepsis immunotherapy.

We thank all authors of this Research Topic, as well as the reviewers for their contributions.

## Author contributions

XL contributed to the drafting of this Editorial. Y-QL made critical revisions and finalized the Editorial. All authors contributed to the article and approved the submitted version.
